# Echocardiographic measurements of right heart pressures in recipients of heart transplant

**DOI:** 10.1002/clc.23873

**Published:** 2022-06-14

**Authors:** Saeed Ghodsi, Mohammadreza Memarjafari, Fatemeh Hadi, Roya Tayeb, Zahra Hosseini

**Affiliations:** ^1^ Department of Cardiology, Sina Hospital Tehran University of Medical Sciences Tehran Iran; ^2^ Research Department, School of Medicine, Tehran Heart Center Tehran University of Medical Sciences Tehran Iran; ^3^ Department of Obstetrics and Gynecology, School of Medicine, Imam Hossein Medical Center Shahid Beheshti University of Medical Sciences Tehran Iran

To the Editor,

We would like to present several ideas and comments regarding “Accuracy of echocardiographic estimations of right heart pressures in adult heart transplant recipients” recently published in a clinical cardiology journal. The authors have examined the accuracy of noninvasive parameters of right side pressures including estimated right atrial pressure (eRAP) and pulmonary artery systolic pressure (PASP) in adults who underwent heart transplants.^1^ They found no correlation between echocardiographic and invasive values of RAP while mean PASP was modestly correlated to average pulmonary pressure determined via right heart catheterization. The authors did not report the frequency of each technical type of heart transplant in the study participants. As two distinct methods in orthotropic heart transplantation are discriminated via partial preservation versus removal of the right atrium, the RA function is expected to be influenced via surgical technique.^2^ In addition, the geometry of the RA in either bicaval anastomoses or the conventional method might affect the RAP and the atrial function. RA area and volume indices may be beneficial in this regard. However, cardiac magnetic resonance imaging determines the atrial structure in an ideal way. There are also multiple echocardiographic parameters for the assessment of the RA pressure. The diameter and respiratory collapse values of inferior vena cava (eRAP IVC), tricuspid E/e′ ratio (eRAP E/e′), and hepatic vein flow (eRAP HV) have been studied. Nevertheless, controversy surrounds the validity of these measures as well as their correlation with invasive values derived from RHC.^3^ Peak systolic and diastolic velocities of hepatic veins are calculated via pulsed wave Doppler and corresponding velocity–time integrals (VTI). Thus, the HV systolic filling fraction is the result of a simple fraction as VTIs/(VTIs + VTId). E/e′ is also a relative measure using inflow velocities of the tricuspid valve.^4^

However, these measures might serve as potential predictors of clinical prognosis as well as correlates of invasive RAP. Furthermore, the authors have not clearly determined the severity of right ventricle dysfunction, which might affect both RAP and PASP in patients. We also suggest adjusting the correlations using Natriuretic peptides with atrial and ventricular origin (Pro‐atrial natriuretic peptide and brain natriuretic peptide) as well as RA volume.
